# ER stress in temozolomide-treated glioblastomas interferes with DNA repair and induces apoptosis

**DOI:** 10.18632/oncotarget.9907

**Published:** 2016-06-07

**Authors:** Jessica L. Weatherbee, Jean-Louis Kraus, Alonzo H. Ross

**Affiliations:** ^1^ Department of Biochemistry and Molecular Pharmacology, University of Massachusetts Medical School, Worcester, Massachusetts, USA; ^2^ Developmental Biology Institute of Marseille-Luminy (IBDML), Aix-Marseille University (AMU) and CNRS, UMR 7288, IBDML, Case 907, Marseille, France

**Keywords:** glioblastoma, endoplasmic reticulum stress, ATF4, apoptosis, DNA double strand breaks

## Abstract

Glioblastoma multiforme (GBM) is a deadly grade IV brain tumor. Radiation in combination with temozolomide (TMZ), the current chemotherapeutic for GBMs, only provides 12–14 months survival post diagnosis. Because GBMs are dependent on both activation of the DNA damage pathway and the endoplasmic reticulum (ER) stress response, we asked if a novel ER stress inducing agent, JLK1486, increases the efficacy of TMZ.

We found that the combination of TMZ+JLK1486 resulted in decreased proliferation in a panel of adherent GBM cells lines and reduced secondary sphere formation in non-adherent and primary lines. Decreased proliferation correlated with increased cell death due to apoptosis. We found prolonged ER stress in TMZ+JLK1486 treated cells that resulted in sustained activation of the unfolded protein response (UPR) through increased levels of BiP, ATF4, and CHOP. In addition, TMZ+JLK1486 treatment caused decreased RAD51 levels, impairing DNA damage repair. Furthermore, we found delayed time to tumor doubling in TMZ+JLK1486 treated mice.

Our data shows that the addition of JLK1486 to TMZ increases the efficaciousness of the treatment by decreasing proliferation and inducing cell death. We propose increased cell death is due to two factors. One, prolonged ER stress driving the expression of the pro-apoptotic transcription factor CHOP, and, second, unresolved DNA double strand breaks, due to decreased RAD51 levels. The combination of TMZ+JLK1486 is a potential novel therapeutic combination and suggests an inverse relationship between unresolved ER stress and the DNA damage response pathway.

## INTRODUCTION

Glioblastoma multiforme (GBM) is an aggressive grade IV brain tumor associated with low patient survival [1]. The current standard of care, comprised of surgical resection, radiation, and the chemotherapeutic agent temozolomide (TMZ), provides the majority of patients with a mere 12 to 14 month survival period post diagnosis [2, 3].

The rapid disease progression and low survival of GBM patients is due to a combination of factors. GBM tumors are highly aggressive and infiltrate into normal brain tissue, making complete surgical resection nearly impossible. Additionally, multidrug resistant pumps in the blood brain barrier (BBB) block chemotherapeutics' access to the brain, limiting the number of effective drugs available to GBM patients [4]. Furthermore, these heterogeneous tumors are in a hypoxic environment [5–7]. This reduces the efficaciousness of radiation and leads to highly resistant cells harboring a variety of protective mutations, allowing them to survive and re-populate the tumor bed. All these factors contribute to inevitable tumor recurrence. There is an urgent need to improve the current standard of care for GBM patients. In order to do so, we asked if the novel combination of an endoplasmic reticulum stress inducer and TMZ enhances efficacy.

TMZ is an oral alkylating DNA agent that efficiently crosses the BBB [8–10]. The majority of DNA alkyl groups, 70% at N7 guanine and 9.2% at N3 adenine, are ultimately not catastrophic as they are repaired by either base excision (BER) or nucleotide excision repair (NER) [10]. However, a small percent of adducts occur on the O6 site of guanine (5%) [10]. Because neither BER nor NER recognizes and excises this alkylated base, these adducts are deleterious to the cell [10]. During replication, the adduct triggers futile mismatch repair, resulting in stalled replication forks and single stranded breaks, which are converted into double stranded breaks (DSBs) during a second round of replication [11, 12]. These DSBs, if not repaired, result in G2/M arrest and eventual cell death [13, 14]. GBM tumors, in particular recurrent ones, circumvent the formation of TMZ-induced DSBs by increasing expression of methyl guanine methyl transferase (MGMT), an enzyme that removes the alkyl group from the O6 guanine site [15]. MGMT restores base integrity, thereby allowing the cell to successfully complete its' cell cycle. Furthermore, detection of the O6 methyl adduct is dependent on a mismatch repair (MMR) response. Cells with mutated MMR proteins do not detect the O6 methyl adduct, allowing propagation of cells with highly damaged genomes. Because GBM cells are dependent on the DNA damage response pathway, treating cells with two agents, one that induces DNA damage (TMZ) and one that inhibits DNA damage repair, may increase tumor cell death [16–19]. To explore this hypothesis, we asked if an endoplasmic reticulum (ER) stress inducer interferes with DNA repair.

JLK1486 is a novel ER stress-inducing agent [20–28]. Although this drug is not electrophilic enough to react with DNA, it can react with thiol residues, interfering with the formation of disulfide bonds essential for tertiary folding of proteins [20]. The resulting accumulation of unfolded and misfolded proteins triggers ER stress and activates the unfolded protein response (UPR) [29, 30]. Initially the UPR is protective; the three receptors that govern UPR, Ire1, ATF6, and PERK, initiate a signaling cascade that increases molecular chaperones, such as BiP/GRP78, while stalling the translation of mRNAs, giving the ER time to resolve this stress [29, 31]. However, prolonged UPR switches from pro-survival to pro-death through upregulation of transcription factors, ATF4 and CHOP, which initiate apoptosis [32–36]. ATF4 is integral to this process as it not only increases CHOP expression during prolonged ER stress, but also promotes autophagy [37]. This initial cytoprotective mechanism becomes cytotoxic if the cell is unable to restore ER homeostasis, emphasizing the vital role of ER-mediated cell survival or cell death. It has been suggested that either blocking ER associated degradation (ERAD) of misfolded proteins or by inducing more ER stress, one may force a switch from pro-survival to pro-apoptosis [31, 38]. Blocking of ERAD in some cancers, such as multiple myeloma, is utilized in the clinic with some success; the FDA approved bortezomib in 2003 for this purpose [39, 40]. However, the use of drugs that inhibit ERAD are limited due to off target toxic effects [41]. Exacerbating ER stress is an attractive alternative. GBMs are solid tumors with cells that survive hypoxia, nutrient deprivation, and low pH. GBM cells have increased BiP/GRP78 levels, suggesting an intrinsic dependence on the ER stress pathway for survival [42]. Interference with the ER stress pathway may be detrimental to cell survival [43, 44].

We tested if the addition of JLK1486 to TMZ increased the efficaciousness of therapy. We reasoned that formation of DNA DSBs occurring in the presence of an overwhelming ER stress response would be catastrophic to cell survival. We found that JLK1486 induces ER stress in GBM cells and when combined with TMZ, reduces proliferation. Decreased proliferation correlated with increased apoptosis. Interestingly, in combination treated cells, we observed decreased RAD51 expression, a key protein for repair of DNA DSBs. We propose reduction of RAD51 levels as the mechanism that accounts for prolonged and unresolved DNA DSBs and increased apoptosis. Combination of JLK1486 with TMZ may provide a potential new chemotherapeutic regimen and, more intriguingly, may link unresolved ER stress with interference of DNA damage repair.

## RESULTS

### JLK1486 is active as a single agent

**Figure 1:**
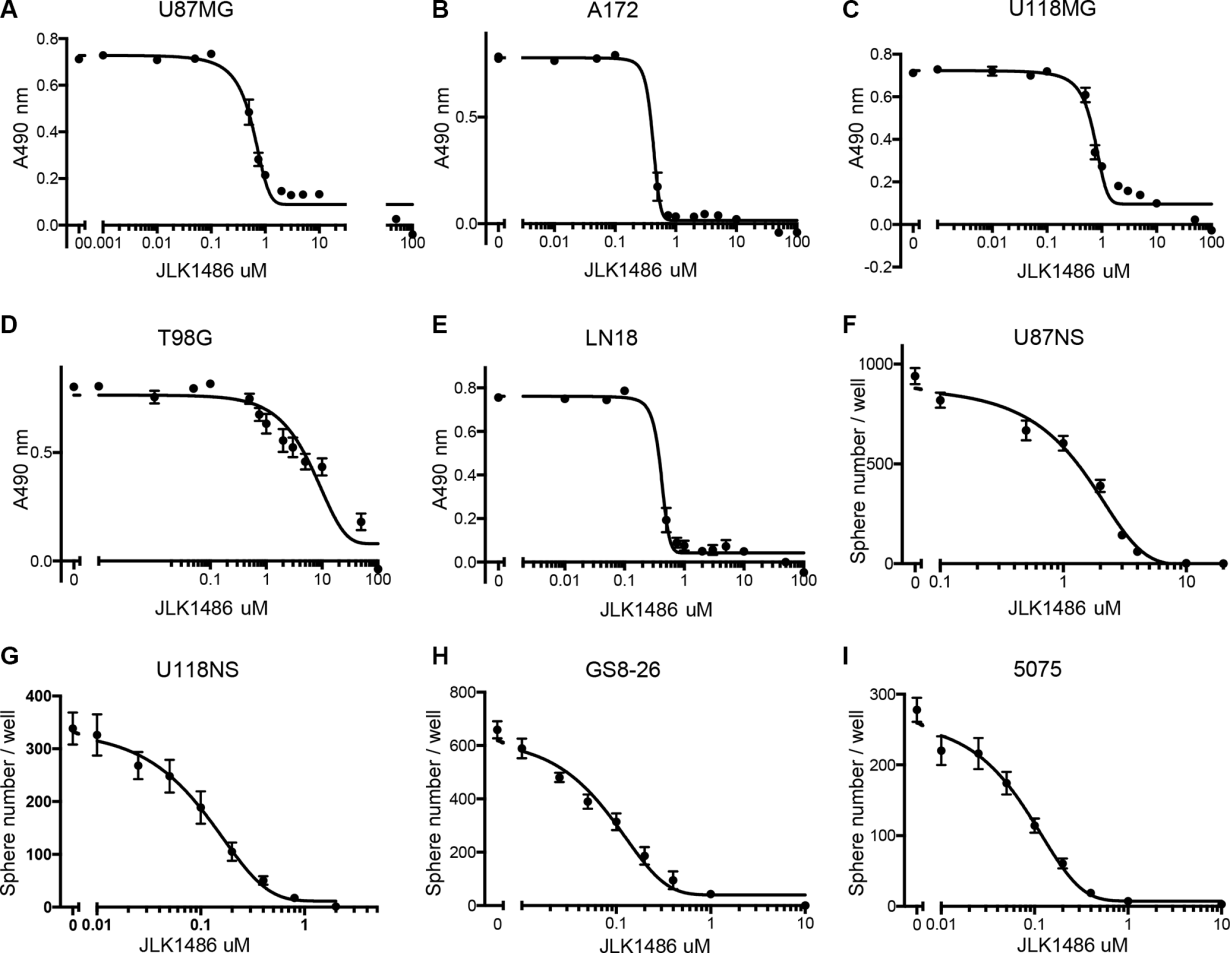
JLK1486 has activity as a single agent (**A–E**) Determination of JLK1486 single agent IC50s in adherent lines via MTS assay. Relative absorbance at 490 nm is shown. (**F–I**) Determination of JLK1486 single agent IC50s in non-adherent and primary lines generated by day 7 neurosphere formation counts. *N* = 3, all error bars are SEM.

To determine the efficacy of JLK1486 as a single agent, we utilized a panel of GBM adherent, non-adherent, and primary lines. For the majority of our established adherent GBM lines, a low concentration of JLK1486 inhibited proliferation (Figure 1A–1E; U87MG IC50 = 0.6 μM; A172 IC50 = 0.26 μM; U118MG IC50 = 0.87 μM; LN18 IC50 = 0.27 μM); however, one line, T98G, was relatively resistant to JLK1486 (Figure 1D; T98G IC50 = 7.6 μM). To assess the efficacy of JLK1486 in both converted non-adherent and primary lines, we employed neurosphere assays in which spheres are dissociated, single cells are plated at clonal density, drug treated, and allowed to grow. On either the seventh (converted non-adherent cell lines) or tenth day (primary cell lines), neurospheres, defined as a single sphere containing ten or more cells, were counted to measure the effects increasing concentrations of JLK1486 had on growth. We found our two converted non-adherent cell lines, U87NS and U118NS, were sensitive to JLK1486 (Figure 1F–1G; U87NS IC50 = 1.6 μM; U118NS IC50 = 0.13 μM). Our primary lines, GS8-26 and 5075, were also sensitive to JLK1486, with an IC50 of 0.08 μM in both lines (Figure 1H–1I).

### JLK1486 combined with TMZ reduces secondary sphere formation more effectively than JLK1486 or TMZ as single agents

**Figure 2:**
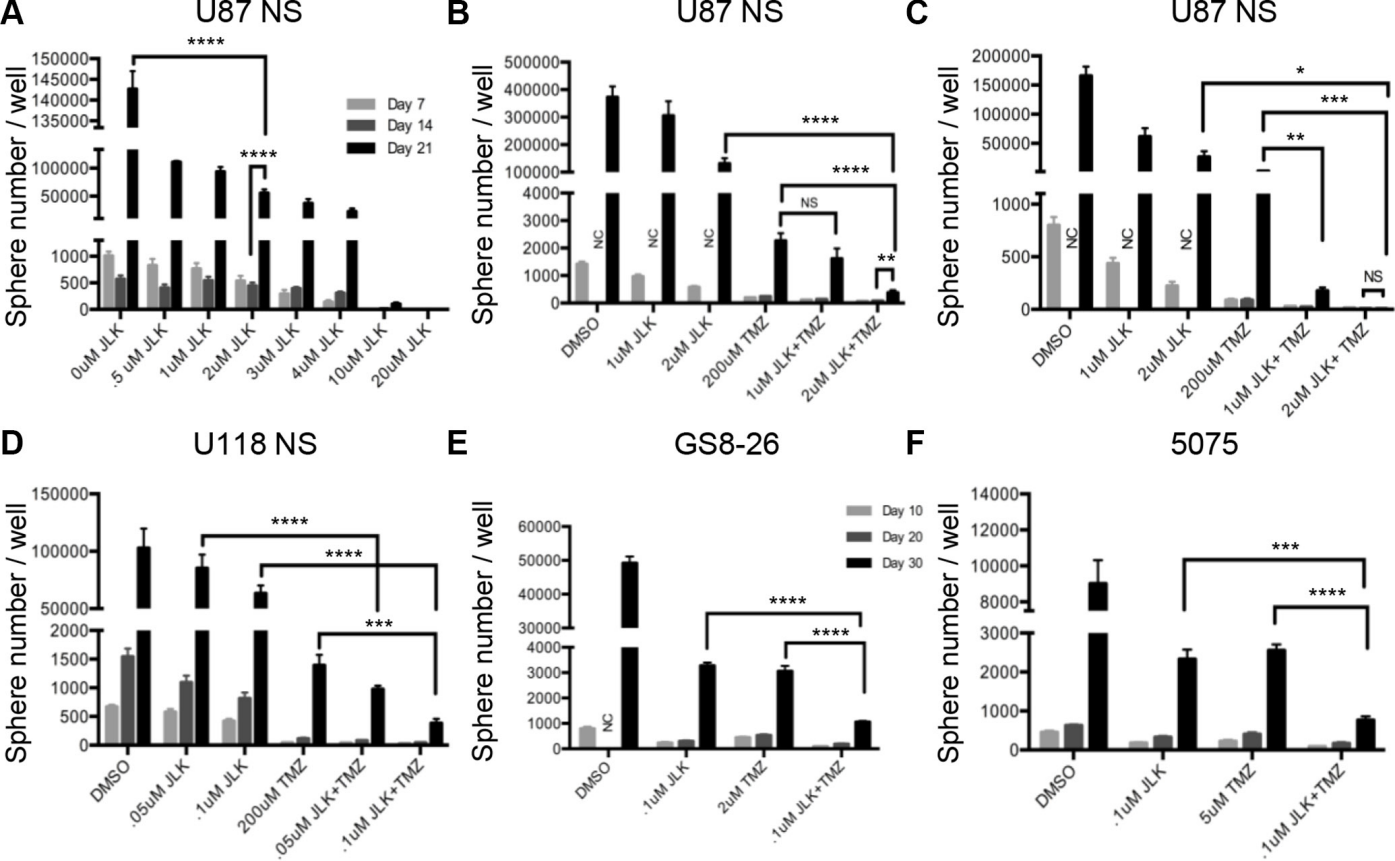
JLK1486 alone does not block secondary sphere formation but when combined with TMZ, secondary sphere formation in decreased (**A**) Secondary sphere formation assay of U87NS cells treated with JLK1486 alone, one time on day 0 (*n* = 3). (**B**) Secondary sphere formation assay of U87NS cells treated with TMZ+JLK1486. Cells were dosed one time on day 0 with both agents (*n* = 4). (**C**) Secondary sphere formation assay of U87NS cells treated with TMZ+JLK1486. Cells were dosed on day 0 with both TMZ+JLK1486 and a second time with JLK1486 on day 7 (*n* = 6). (**D**) Secondary sphere formation of U118NS cells treated with TMZ+JLK1486 on day 0 and a second time with JLK1486 on day 7 (*n* = 3). (**E**) Secondary sphere formation of primary line GS8-26 cells treated with both TMZ+JLK1486 on day 0 and with JLK1486 on day 7 (*n* = 3). (**F**) Secondary sphere formation of primary line 5075 cells treated with both TMZ+JLK1486 on day 0 and JLK1486 on day 7 (*n* = 3). NC = not counted because neurospheres were too numerous. All error bars are SEM, two-tailed *t*-test, *P = 0.01, **P = 0.001-0.007,***P = 0.0002-0.0005,****P < 0.0001.

Secondary sphere formation assays are an *in vitro* tool to mimic the clinical recurrence universally exhibited in GBM patients. Cell lines are dissociated, plated at clonal densities, drug treated and allowed to grow for seven or ten days. Fresh medium is added on day seven (U87NS, U118NS) or ten (GS8-26, 5075), and cells are allowed to grow an additional seven (U87NS, U118NS) or ten (GS8-26, 5075) days, and then counted, allowing cell and sphere recovery to be assessed. On day fourteen (U87NS, U118NS) or day twenty (GS8-26, 5075), spheres are dissociated to single cells, re-plated, allowed to grow for an additional seven (U87NS, U118NS) or ten days (GS8-26, 5075), and then counted to assess secondary sphere formation (Supplementary Figure 1A–1B). To determine if JLK1486 as a single agent was capable of blocking secondary sphere formation, we carried out a neurosphere formation assay with U87NS cells using a range of JLK1486 doses from 0 μM to 20 μM. Although JLK1486 alone at the IC50 for U87NS (2 μM) (Figure 2A) did not completely block secondary sphere formation, there was a statistically significant reduction of day 21 secondary spheres compared to the DMSO control sample. A higher dose of JLK1486 (20 μM, ten times higher than the IC50) completely blocked secondary sphere formation (Figure 2A). Reduced sphere formation suggests that JLK1486 may be a novel chemotherapeutic for decreasing recurrence.

This led us to ask if the efficacy of TMZ, the chemotherapeutic agent currently used in the clinic, could be improved if used in combination with JLK1486. We performed secondary sphere formation assays using TMZ alone (the relevant dose of TMZ in our converted non-adherent lines has been previously described [45]) and in combination with a sub-IC50 dose of JLK1486 (1 μM) as well as the IC50 dose (2 μM) in U87NS cells (Supplementary Figure 1C). We did not find a statistically significant reduction of secondary spheres for the sub-optimal dose of TMZ+ 1 μM JLK1486 when compared to TMZ alone (Figure 2B). We did find significant reduction of secondary sphere formation in TMZ + 2 μM JLK1486 versus TMZ or JLK1486 alone (Figure 2B). However, there was not a complete block in secondary sphere formation in the TMZ+2 μM JLK1486 dose, indicated by the statistically significant increase in sphere formation in day 14 versus day 21 samples (Figure 2B).

In the clinic GBM patients receive multiple doses of chemotherapeutics [2, 3]. We asked if two doses of JLK1486 would increase the efficaciousness of the TMZ+JLK1486 combination treatment. We carried out secondary sphere formation assays in which cells were dosed with TMZ+JLK1486 on day 0 and then treated a second time with JLK1486 alone on day 7 (Supplementary Figure 1D). We found significant secondary sphere reduction in the sub-optimal dose combination of TMZ+ 1 μM JLK1486 2X versus TMZ+ 1 μM JLK1486 1X (Figure 2C versus Figure 2B) as well as inhibition of secondary sphere formation in TMZ+ 2 μM JLK1486 2X versus TMZ+ 2 μM JLK1486 1X (Figure 2C versus Figure 2B). Additionally, we found significantly decreased secondary sphere formation in our converted non-adherent U118NS line as well as our primary lines GS8-26 and 5075 when cells were treated on day 0 with TMZ+ JLK1486 and a second time with JLK1486 on day 7 (Figure 2D–2F). This demonstrates that the TMZ+ JLK1486 2X is an effective combination therapy to decrease secondary sphere formation and may be a schedule-dependent process. All further experiments were conducted using TMZ+JLK1486 2X.

### TMZ+JLK1486 treatment results in decreased cell growth and increased cell death in U87NS

**Figure 3:**
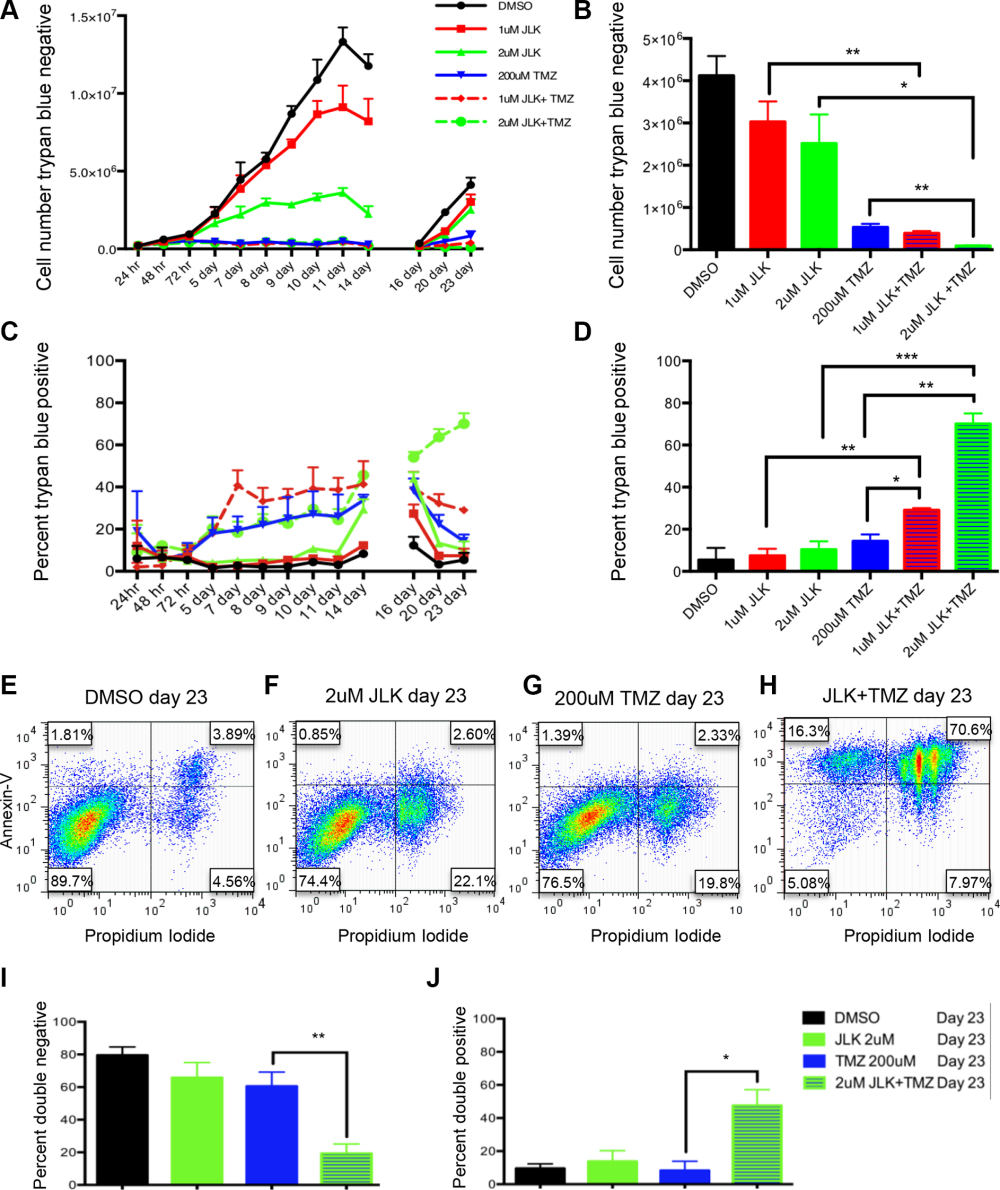
TMZ+JLK1486 double treatment results in decreased cell growth and increased cell death in U87NS cells (**A**) The number of trypan blue negative cells in U87NS cells treated with DMSO, 1 μM JLK1486, 2 μm JLK1486, 200 μM TMZ, TMZ + 1 μM JLK1486, and TMZ + 2 μm JLK1486 collected over 24 hours to 23 days (*n* = 3). (**B**) Quantification of the number of trypan blue negative U87NS cells in DMSO, 1 μM JLK1486, 2 μm JLK1486, 200 μM TMZ, TMZ + 1 μM JLK1486, and TMZ + 2 μm JLK1486 conditions at day 23 (*n* = 3). (**C**) The percent of trypan blue positive cells in U87NS cells treated with DMSO, 1 μM JLK1486, 2 μm JLK1486, 200 μM TMZ, TMZ +1 μM JLK1486, and TMZ + 2 μm JLK1486 collected over a 24 hour to 23 day time course (*n* = 3). (**D**) Quantification of the percent of trypan blue positive U87NS cells in DMSO, 1 μM JLK1486, 2 μm JLK1486, 200 μM TMZ, TMZ + 1 μM JLK1486, and TMZ +2 μm JLK1486 conditions at day 23 (*n* = 3). (**E–H**) Representative FACs analysis comparing propidium versus annexin V staining of day 23 U87NS cells treated with DMSO, 2 μm JLK1486, 200 μM TMZ, and TMZ+2 μm JLK1486 (*n* = 4). (**I**) Percent of PI and Annexin V double negative cells in day 23 U87NS cells treated with DMSO, 2 μm JLK1486, 200 μM TMZ, and TMZ + 2 μm JLK1486 (*n* = 4). (**J**) Percent of PI and Annexin V double positive cells in day 23 U87NS cells treated with DMSO, 2 μm JLK1486, 200 μM TMZ, and TMZ + 2 μm JLK1486 (*n* = 4). All error bars are SEM, two-tailed *t*-test, *P = 0.01–0.02, **P = 0.001–0.008, ***P = 0.0007.

To determine how TMZ+2 μM JLK1486 treatment reduced secondary sphere formation in U87NS cells, we carried out a time course ranging from 24 hours to 23 days to evaluate the number of trypan blue positive and negative cells. Control cells treated with DMSO had the highest rate of proliferation from day 0 to day 14 (Figure 3A). Cells treated with either 1 μM or 2 μM JLK1486 increased in number from day 0 to day 14, however, there were significantly fewer JLK1486-treated cells versus the DMSO control (Figure 3A). Cells treated with either TMZ alone or TMZ in combination with 1 μM or 2 μM JLK1486 did not undergo significant proliferation from day 0 to day 14 (Figure 3A). This was expected as it has been well established in the literature that TMZ induces DNA double strand breaks that result in G2/M arrest. After day 14 dissociation and re-plating, DMSO, 1 μM and 2 μM JLK1486-treated cells, as well as TMZ alone treated cells underwent significant proliferation from day 16 to day 23 (Figure 3A–3B). Although the number of TMZ+1 μM JLK1486-treated cells was less than TMZ alone treated cells on day 23, this was not statistically significant (Figure 3B). However, cells treated with TMZ+2 μM JLK1486 were incapable of repopulating their cultures and maintained a statistically significant reduction in cell number versus JLK1486 alone as well as TMZ alone (Figure 3B). This suggests that inhibition of secondary sphere formation in TMZ+2 μM JLK1486-treated cells is at least partly the result of treated cells' inability to proliferate.

We simultaneously collected trypan-blue-positive counts to detect cell death in U87NS control, single agent, and TMZ+JLK1486-treated cells. We observed significant increases in cell death in TMZ alone as well as TMZ+1 μM JLK1486 and TMZ+2 μM JLK1486-treated cells versus DMSO and 1 μM and 2 μM JLK1486 single-treated cells in the first half of our time course (Figure 3C; days 0-14). However, post day 14, we observed a significant decrease in the percentage of trypan blue positive cells from day 16 to day 23 in TMZ alone treated cells (Figure 3C). Although the percentage of trypan-blue-positive TMZ+1 μM JLK1486 cells also decreased for samples post day 14, the percent remained significantly higher than TMZ or 1 μM JLK1486 alone (Figure 3C). Conversely, the percent of trypan-blue-positive cells in TMZ+2 μM JLK1486-treated cells continued to increase post day 14 (Figure 3C). This resulted in a 70.0% (+/− 5.0) trypan-blue-positive population in TMZ+ 2 μM JLK1486-treated cells versus 14.3% (+/− 3.2) in TMZ-treated cells and 10.3% (+/− 3.9) in 2 μM JLK1486-treated cells (Figure 3D).

To test if the observed increase in cell death was due to apoptosis, we performed FACS analysis with annexin V and propidium iodide (PI) staining of 23-day samples. The annexinv V and PI staining corroborated our trypan blue counts as we observed 70.6% double positive cells in TMZ + 2 μM JLK1486-treated cells versus 3.9% in DMSO, 2.6% in 2 μM JLK1486 alone, and 2.3% in TMZ alone treated cells (Figure 3E–3H; 3I–3J). This demonstrates that TMZ + 2 μM JLK1486 treatment in U87NS cells results in reduced cell growth due to the induction of apoptosis.

### TMZ+JLK1486 treatment induces prolonged endoplasmic reticulum stress that results in induction of CHOP, a pro-apoptotic transcription factor

It is well established that prolonged, unresolved ER stress triggers apoptosis [32–34]. To determine if TMZ + 2 μM JLK1486 treatment results in prolonged ER stress induction, we collected a series of protein lysates over a 24-hour to 23-day time course. We probed protein lysates for levels of BiP, a key heat shock molecular chaperone indicative of ER stress, as well as ATF4, a transcription factor that initially serves as a pro-survival signal but switches to pro-apoptotic when ER stress is unresolved. ATF4 drives increased expression of the pro-apoptotic transcription factor CHOP. We therefore analyzed protein lysates for ATF4 and CHOP to detect this switch.

**Figure 4:**
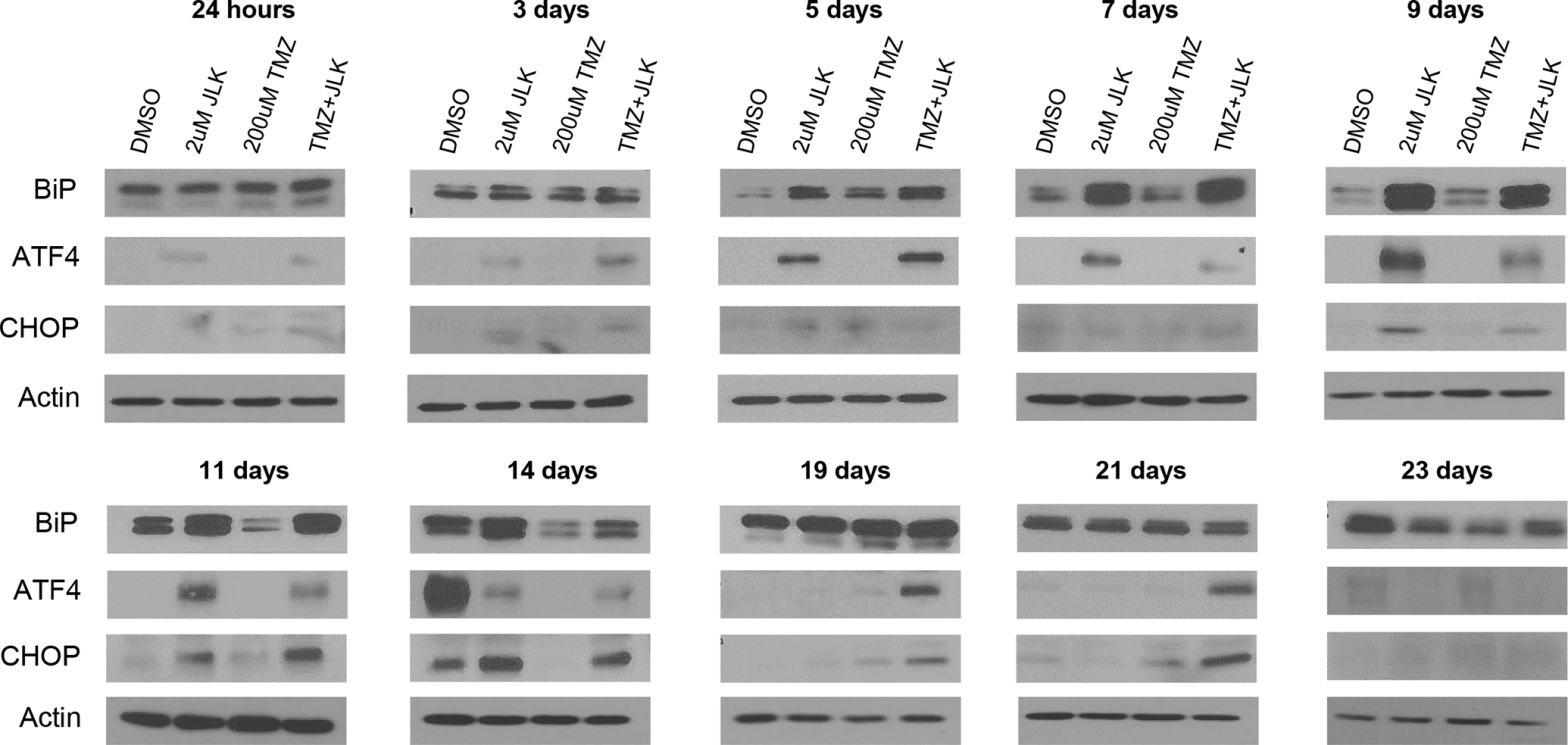
TMZ+JLK1486 treatment induces prolonged endoplasmic reticulum stress that results in induction of CHOP, a pro-apoptotic transcription factor Western blot analysis of ER stress response factors, BiP, ATF4, and CHOP in whole cell U87NS lysates harvested after 24 hours to 23 days treatment of DMSO, 2 μM JLK1486, 200 μM TMZ, and TMZ + 2 μM JLK1486. Blots are representatives of *n* = 3.

In 2 μM JLK1486 and TMZ + 2 μM JLK1486-treated cells we observed increased expression of BiP that was maintained 14 days post treatment, suggesting that JLK1486 induces prolonged ER stress (Figure 4). For post treatment day 14, BiP levels were highly elevated in all conditions (Figure 4). Increased expression of ATF4 was observed only in JLK1486 and TMZ + 2 μM JLK1486-treated cells (Figure 4). Induction began three days post treatment and was maintained 21 days post treatment, suggesting generation of long-term ER stress (Figure 4). We did find strong expression of ATF4 in day 14 DMSO-treated cells (Figure 4). We suggest this induction is due to nutritional deprivation resulting from these rapidly proliferating cells becoming overgrown. This is substantiated by a slight decrease in day 14 trypan blue negative cell number (Figure 3A) as well as the lack of increased and sustained CHOP induction of day 14, 19, and 21 DMSO versus 2 μM JLK1486 alone or TMZ + 2 μM JLK1486-treated samples (Figure 4). Induction of CHOP was not observed until 9 days post 2 μM JLK1486 and TMZ + 2 μM JLK1486 treatment (Figure 4). CHOP levels were maintained until day 14 in 2 μM JLK1486 alone and TMZ + 2 μM JLK1486-treated cells. Increased CHOP expression was detected in TMZ + 2 μM JLK1486-treated cells in day 19 and day 21 lysates (Figure 4). ATF4 and CHOP were undetectable in all day 23 protein lysates (Figure 4). Induction of BiP and ATF4 in 2 μM JLK1486 and TMZ + 2 μM JLK1486-treated cells suggests that JLK1486 is an effective ER stress-inducing agent and may promote cell death via prolonged ATF4 expression driving CHOP in TMZ + 2 μM JLK1486-treated cells.

### TMZ+JLK1486 treatment triggers prolonged activation of DNA damage response pathway and promotes unresolved DNA double stand breaks

TMZ induces the formation of DNA DSBs. This results in phosphorylation of DNA damage sensors, ATM and CHK2, which in turn induces phosphorylation of H2A.X, a key marker for DSBs, and recruitment of RAD51 to DSBs to initiate homologous recombination [46–48]. To determine if the combination of TMZ + 2 μM JLK1486 increases and/or prolongs DNA damage, we analyzed a series of protein lysates collected from 24 hours to 23 days post treatment for P ATM, ATM, P CHK2, CHK2, RAD51, and γH2A.X.

**Figure 5:**
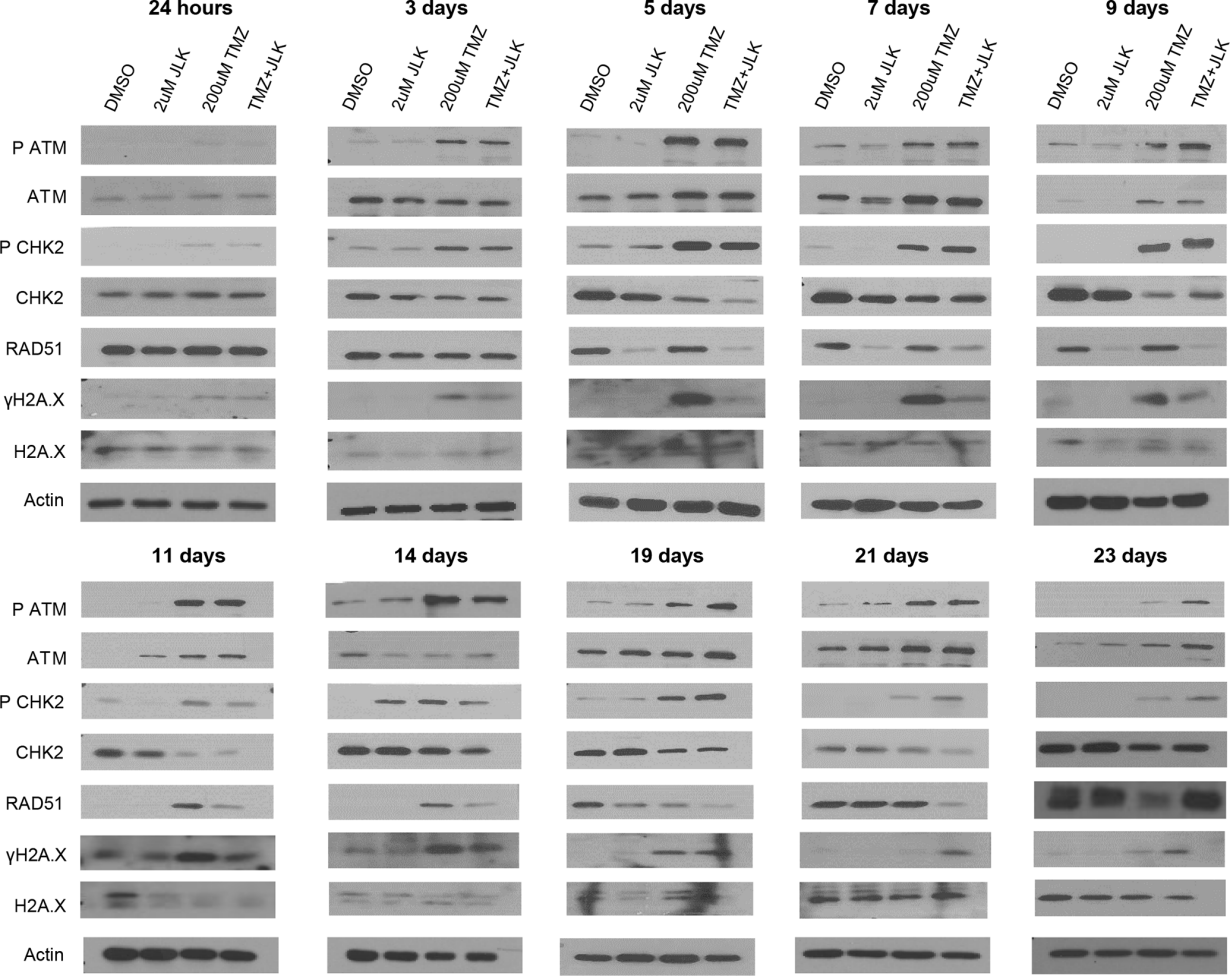
TMZ+JLK1486 treatment triggers prolonged activation of DNA damage response pathway and promotes unresolved DNA double stand breaks Western blot analysis of DNA damage sensors (P ATM, ATM, P CHK2, CHK2) and markers for DNA DSBs (RAD51, ΥH2A.X) from whole cell U87NS lysates harvested after 24 hours to 23 days of DMSO, 2 μM JLK1486, 200 μM TMZ, and TMZ + 2 μM JLK1486 treatment. Blots are representatives of *n* = 3.

We observed phosphorylation of ATM and CHK2 24 hours post treatment in TMZ and TMZ + 2 μM JLK1486-treated cells (Figure 5). Increased levels of P ATM and P CHK2 were maintained in TMZ and TMZ + 2 μM JLK1486-treated cells throughout the time-course, however, we noted higher levels of P ATM and P CHK2 in post day 14 combination lysates, suggesting TMZ + 2 μM JLK1486 treatment results in a sustained DNA damage response (Figure 5). Additionally, we detected extended phosphorylation of H2A.X in TMZ + 2 μM JLK1486-treated cells, suggesting substantially more unresolved DNA DSBs in combination versus TMZ single treated cells (Figure 5; Supplementary Figure 2). Although high levels of RAD51 were initially observed in all conditions, we found RAD51 levels decreased 5 days post treatment in 2 μM JLK1486 alone and TMZ + 2 μM JLK1486-treated cells and were continually lower than TMZ alone treated cells until 21 days post treatment (Figure 5). Increased expression of RAD51 was not detected until 23 days post treatment. Detection of increased P ATM, P CHK2, and prolonged γH2A.X in TMZ + 2 μM JLK1486 cells suggests that combination treatment not only prolongs the DNA damage response, but also promotes unresolved DNA DSBs over an extended time course through reduction of RAD51.

### Knockdown of ATF4 does not rescue secondary sphere formation but does decrease cell death in TMZ+JLK1486 treated cells

**Figure 6:**
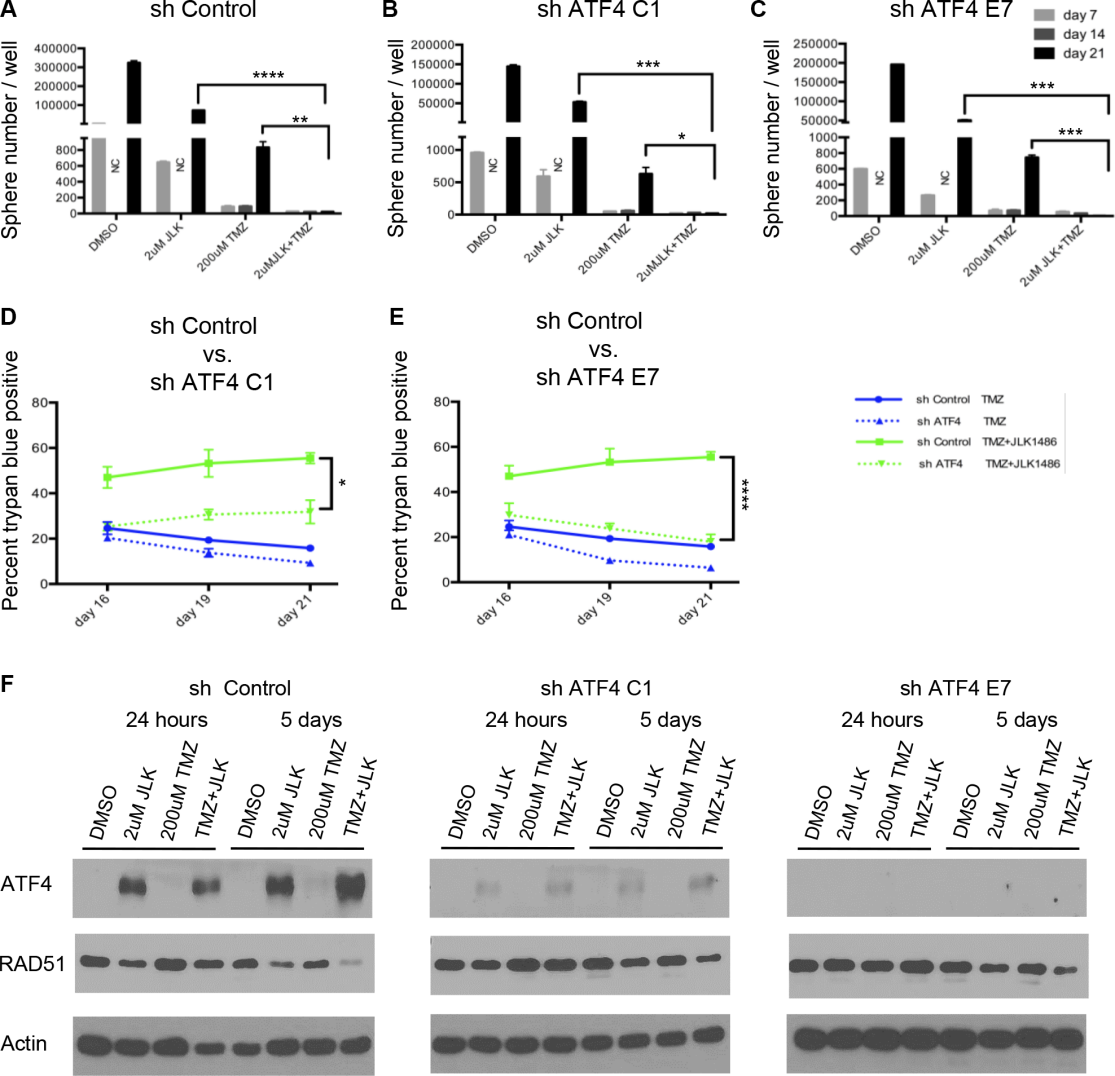
Knockdown of ATF4 does not rescue secondary sphere formation but does decrease cell death in TMZ+JLK1486 treated cells (**A**) Secondary sphere formation of U87NS sh control cells treated with both TMZ+2 μM JLK1486 on day 0 and with 2 μM JLK1486 on day 7 (*n* = 3). (**B**) Secondary sphere formation of U87NS shATF4 C1 cells treated with both TMZ+2 μM JLK1486 on day 0 and with 2 μM JLK1486 on day 7 (*n* = 3). (**C**) Secondary sphere formation of U87NS shATF4 E7 cells treated with both TMZ+2 μM JLK1486 on day 0 and with 2 μM JLK1486 on day 7 (*n* = 3). (**D**) Percent of trypan blue positive cells in U87NS sh control versus shATF4 C1 after 16, 19, and 21 days of 200 μM TMZ versus TMZ+2 μM JLK1486 treatment (*n* = 3). (**E**) Percent of trypan blue positive cells in U87NS sh control versus shATF4 E7 after 16, 19, and 21 days of 200 μM TMZ versus TMZ + 2 μM JLK1486 treatment (*n* = 3). (**F**) Western blot analysis of RAD51 and ATF4 protein extracted from whole cell U87NS sh control, U87NS shATF4 C1, and U87NS shATF4 E7 cells treated with DMSO, 2 μM JLK1486, 200 μM TMZ, TMZ + 2 μM JLK1486 for either 24 hours or 5 days. NC = not counted because neurospheres too numerous. Representative blot shown (*n* = 3). All error bars are SEM, two-tailed *t*-test, *P = 0.03,**P = 0.008, ***P = 0.001-0.003,****P = 0.0001–0.0007.

Because we observed inhibition of secondary sphere formation (Figure 2), increased cell death (Figure 3) and prolonged expression of ATF4 in TMZ + 2 μM JLK1486-treated cells (Figure 4), we asked if knockdown of ATF4 would rescue secondary sphere formation and decrease cell death. To determine this, we generated three stable U87NS lines, one expressing an shRNA control, and two lines expressing shRNAs against ATF4, shATF4 C1 and shATF4 E7. The U87NS sh control, shATF4 C1, and shATF4 E7 lines were treated with DMSO, 2 μM JLK1486, 200 μM TMZ, and TMZ + 2 μM JLK1486, protein lysates were collected at 24 hours and five days post treatment, and ATF4 levels were examined via western. Because neurosphere and trypan blue assays were carried out with cells plated at passage four and assays completed by passage six, we analyzed ATF4 expression levels in our knockdown lines at passage number six to verify that knockdown was maintained throughout the experimental time-course. We observed robust induction of ATF4 in 2 μM JLK1486 and TMZ + 2 μM JLK1486 sh control treated U87NS cells, slight induction of ATF4 in 2 μM JLK1486 and TMZ + 2 μM JLK1486 shATF4 C1 treated U87NS cells, and no expression of ATF4 in 2 μM JLK1486 and TMZ + 2 μM JLK1486 shATF4 E7 treated U87NS cells (Figure 6F).

To determine if knockdown of ATF4 rescues secondary sphere formation, we treated our sh control, shATF4 C1, and shATF4 E7 U87NS lines with DMSO, 2 μM JLK1486, 200 μM TMZ, and TMZ + 2 μM JLK1486 and carried out neurosphere assays. On day 21, we did not observe formation of secondary spheres in the U78NS sh control or in either of our U87NS shATF4 lines, C1 or E7, demonstrating that knockdown of ATF4 does not rescue secondary sphere formation in TMZ + 2 μM JLK1486-treated cells (Figure 6A–6C).

As neurosphere assays may not evaluate the effect drug treatment has on viability, we determined if ATF4 knockdown decreased cell death in our U87NS sh control and shATF4 C1 and E7 lines by carrying out a time course of trypan blue counts. As increased cell death in U87NS TMZ +2 μM JLK1486-treated cells was most significant at later time points (Figure 3), we focused on analyzing the number of trypan-blue-positive cells in our control and ATF4 knockdown lines at day 16, 19, and 21 time points. Furthermore, because U87NS cells treated with TMZ alone had significant reduction in cell growth, but were able to repopulate the culture versus TMZ + 2 μM JLK1486-treated cells (Figure 3B), we were most interested in comparing the effects of ATF4 knockdown in TMZ versus TMZ + 2 μM JLK1486-treated cells (all conditions shown in Supplementary Figure 3).

We observed a statistically significant decrease in the number of trypan-blue-positive cells in ATF4 knockdown versus sh control TMZ + 2 μM JLK1486-treated cells at day 19 and day 21 (Figure 6D–6E; Day 19: C1 = 31%; E7 = 24%; control = 53%) (Figure 6D–6E; Day 21: C1 = 32%; E7 = 18%; control = 56%). Reduction of cell death in ATF4 U87NS knockdown cells treated with TMZ + 2 μM JLK1486 suggests ATF4 may play a role in promoting cell death in TMZ + 2 μM JLK1486-treated cells.

To explore why ATF4 knockdown results in decreased cell death in TMZ + 2 μM JLK1486-treated cells, we analyzed levels of RAD51 in sh control versus shATF4 U87NS treated with DMSO, 2 μM JLK1486, 200 μM TMZ, and TMZ + 2 μM JLK1486. We analyzed lysates collected 24 hours and 5 days post treatment as we saw decreased expression of RAD51 in 2 μM JLK1486 and TMZ + 2 μM JLK1486 U87NS treated cells at day 5 (Figure 5). Interestingly, we observed increased RAD51 levels in shATF4 knockdown lines versus sh control U87NS cells after 5 days of TMZ + 2 μM JLK1486 treatment (Figure 6F). This suggests a potential inverse relationship between ER stress induction of ATF4 and RAD51 protein levels.

### TMZ+JLK1486 treatment delays tumor doubling *in vivo*

**Figure 7:**
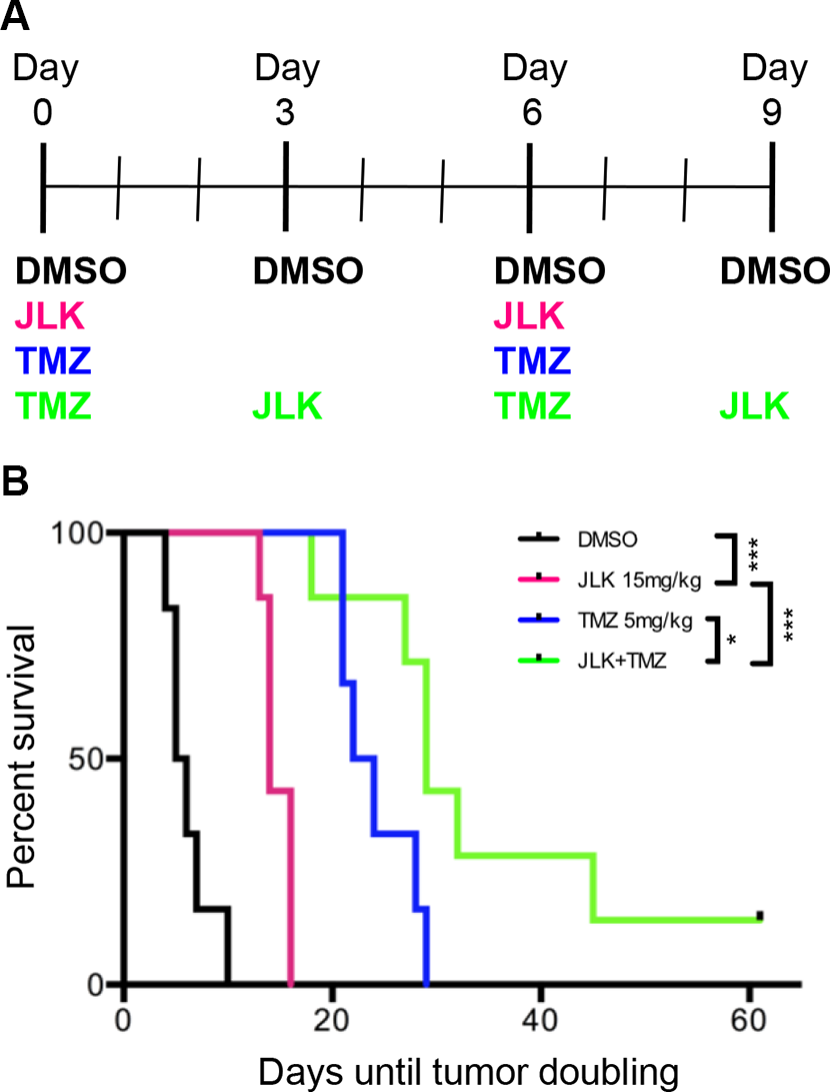
TMZ+JLK1486 treatment delays tumor doubling *in vivo* (**A**) Dosing schedule implemented for NU/NU mice intraperitoneal (IP) injected with DMSO vehicle, JLK1486 15mg/kg, TMZ 5 mg/kg or both drugs. (**B**) Kaplein-Meier survival curve comparing time to tumor doubling in DMSO, JLK1486 15mg/kg, TMZ 5 mg/kg, and combination treated NU/NU mice. JLK1486 vs. DMSO ***P = 0.0002; TMZ vs. DMSO *P* = 0.0005; TMZ+JLK1486 vs. DMSO *P* = 0.0002; TMZ vs. JLK1486 *P* = 0.0007; TMZ+JLK1486 vs. JLK1486 ***P = 0.0003; TMZ+JLK1486 vs. TMZ *P = 0.04. Statistics generated via a log-rank test.

To determine if the combination of TMZ+JLK1486 is effective *in vivo*, we subcutaneously injected nude mice with U87NS cells, allowed tumors to form, and treated with DMSO, JLK1486 15 mg/kg, TMZ 5 mg/kg, or TMZ with JLK1486 (Figure 7A). We used time to tumor volume doubling as our readout to compare control, single agent, and double agent treated mice.

We found significant delay in tumor doubling in JLK1486 versus DMSO (*p* = 0.0002), TMZ versus DMSO (*p* = 0.0005), TMZ versus JLK1486 (*p* = 0.0007), TMZ+JLK1486 versus JLK1486 (*p* = 0.0003), and TMZ+JLK1486 versus TMZ alone (*p* = 0.04) treated mice (Figure 7B). This significant delay in tumor volume doubling for TMZ+JLK1486-treated mice suggests the combination should be further studied as it may have clinical applications.

## DISCUSSION

There is an urgent need to improve the current chemotherapy for GBM patients. We determined if the addition of a novel ER stress inducer, JLK1486, would increase the efficaciousness of TMZ treatment. We reasoned that targeting two different pathways essential to tumor survival would inhibit GBM cell proliferation and promote cell death. We found that when GBM cells were treated with TMZ+JLK1486, we were able to reduce secondary sphere formation and in the case of U87NS cells, completely block secondary sphere formation, suggesting that this combination is effective at inhibiting tumor cells from re-populating their culture. This is an important finding as GBM therapies fail due to tumor recurrence [2, 3]. The mechanism of secondary sphere inhibition in U87NS cells is increased cell death. Interestingly, this effect was maintained over an extended time course, suggesting the combination provides a long-term effect. Furthermore, we found that treatment of subcutaneous tumors in mice with TMZ+JLK1486 significantly delayed tumor doubling, suggesting the potential use of the combination in a clinical setting. We propose two models by which TMZ+JLK1486 promote cell death. One in which prolonged, unresolved ER stress drives apoptosis and one in which the accumulation of unrepaired, deleterious DNA double strand breaks triggers apoptosis.

To understand the mechanism driving the enhanced efficacy observed in TMZ+JLK1486 U87NS treated cells we delineated the effects the combination exerted on the ER stress response pathway, with particular attention to levels of ATF4 and CHOP induction. It is well established that GBM cells are reliant upon the ER stress pathway and that overwhelming the ER stress pathway switches the initial pro-survival response to one of pro-death. JLK1486 is a viable candidate for this as it prevents the formation of disulfide bonds that are essential for protein folding and functionality. Indeed, when U87NS cells are treated with JLK1486 we see induction of ATF4 and its downstream target, CHOP, in JLK1486-treated cells, not TMZ-treated cells. This validates our hypothesis that JLK1486 and TMZ target different pathways and reinforces the reasoning for why this dual treatment provides a robust response.

We noted a decrease in ATF4 levels in day 7 JLK1486 alone and TMZ+JLK1486 protein lysates. We found this intriguing as our initial drug regiment, (Supplementary Figure 1C) utilizing only one dose (1X) of JLK1486 at day 0 in combination with TMZ did not result in U87NS secondary sphere inhibition (Figure 2B). However, when we added a second dose (2X) of JLK1486 at day 7, we observed complete inhibition of U87NS secondary sphere formation (Supplementary Figure 1D; Figure 2C). It is plausible the second dose of JLK1486 at day 7 enhances inhibition of sphere formation by maintaining increased ATF4 levels that contribute to a sustained and unresolved ER stress response. The expression levels of ATF4 correlates with increased expression of its downstream target, CHOP, further strengthening this model. Increased levels of CHOP, a driver of apoptosis, are not observed until after the second dose of JLK1486 on day 7, again suggesting the second dose of JLK1486 is necessary to prolong ER stress levels and force the pro-survival to pro-apoptotic switch that contributes to reduced secondary spheres and cell death (Figure 2C; Figure 3; Figure 4). Sustained ATF4 and CHOP expression in TMZ+JLK1486-treated cells correlates with our trypan blue positive time course, which shows increased cell death post day 14 (Figure 3C–3D). This suggests a model in which TMZ+JLK1486 treatment initiates, maintains, and promotes unresolved ER stress that drives apoptosis.

**Figure 8:**
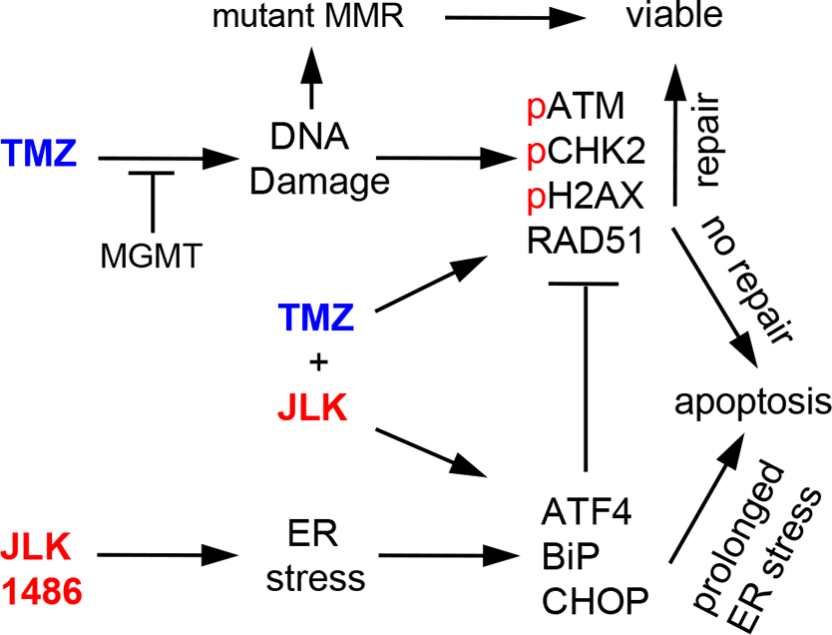
TMZ+JLK1486 treatment induces prolonged ER stress and unresolved DNA damage that results in increased cell death TMZ activates the DNA damage response pathway due to the generation of DNA double strand breaks. If the breaks are repaired, the cells survive; if not, cells undergo apoptosis. JLK1486 induces ER stress. If ER stress is resolved, cells survive; if not, cells undergo apoptosis through ATF4 mediated up-regulation of CHOP. TMZ+2 μM JLK1486 treatment prolongs ER stress, activates the DNA damage response, and causes unresolved DNA DSBs that result in apoptosis due to decreased RDA51 levels, possibly mediated by ER stress induced ATF4 expression. GBM cells generate resistance to TMZ through several mechanisms, including increased expression of methyl guanine methyl transferase (MGMT), which removes alkyl groups from O6 guanine residues, and mutations of the mismatch repair (MMR) system, allowing aberrant cells to enter and complete the cell cycle.

TMZ treatment results in the formation of DNA DSBs. If these breaks are not repaired, cells undergo apoptosis. Both TMZ alone and TMZ+JLK1486-treated samples show induction of DNA damage at early and late time-points; however, TMZ+JLK1486-treated samples exhibit stronger activation at later time points (Figure 5), suggesting prolonged DNA damage. Although both TMZ alone and TMZ+JLK1486 samples exhibit markers for unresolved DNA DSBs, only TMZ-treated cells have increased expression of RAD51, a key protein required for repair of DSBs. RAD51-mediated repair of DSBs would lead to cell survival and proliferation. We find this to be true in our TMZ alone treated cells where trypan blue positive counts decrease and trypan blue negative counts increase over time (Figure 3). Conversely, the decreased levels of RAD51 observed in TMZ+JLK1486-treated cells would lead to accumulation of un-resolved DNA DSBs, prolonged γH2A.X induction and increased cell death. This pattern is exhibited in TMZ+JLK1486-treated samples where RAD51 levels are substantially lower than TMZ alone treated samples, correlating with increased DNA DSBs (Figure 5; Supplementary Figure 3). Accumulation of un-resolved DNA DSBs due to decreased RAD51 levels is a plausible second mechanism for why TMZ+JLK1486-treated cells are unable to re-populate and instead initiate apoptosis (Figure 3H; Figure 5; Figure 8).

As we suggest two possible mechanisms that account for induction of cell death in TMZ+JLK1486-treated cells, one due to prolonged ER stress and the second due to unresolved DNA DSBs, we asked if a link between ER stress induction and RAD51 protein reduction could be found. When ATF4 is strongly expressed in JLK1486 and TMZ+JLK1486-treated samples, we observed reduction of RAD51 protein levels (Figure 4; Figure 5; Figure 6F). Conversely, when ATF4 levels decrease over time, as in day 23 samples (Figure 4) or are reduced via shRNA (Figure 6), RAD51 increases (Figure 5; Figure 6F) This suggests a potential novel inverse relationship where ER stress, possibly through ATF4 induction, decreases RAD51 levels (Figure 8). Although the potential mechanism of how ATF4 may regulate RAD51 expression is beyond the scope of this study, one could envision several possibilities. Perhaps ATF4 itself acts as a repressor of RAD51 transcription or drives the expression of RAD51 repressors or miRs that negatively regulate RAD51 translation. Nonetheless, it may serve as a novel interaction to be explored. Supporting this, Yamamori et al reported that ER stress in lung carcinoma cells leads to enhanced proteasomal degradation of Rad51 [43]. In conclusion, we suggest that TMZ+JLK1486 is an effective novel drug combination that results in cell death of U87NS cells due to the combination of prolonged ER stress induction and the inability to resolve DNA damage through RAD51 reduction. As this study was being prepared for publication, Xipell et al proposed a similar model by which ER stress suppresses multiple DNA repair proteins [49].

## MATERIALS AND METHODS

### Cell lines and cell culture reagents

U87MG, A172, T98G, and LN18 cell lines were purchased from American Type Culture Collection (ATCC). U118MG cells were a kind gift from the laboratory of Dr. Larry Recht (Stanford University, Stanford, CA, 2003). Cell lines were verified via the Radil Idexx Cell Check (9 short tandem repeats) and maintained as monolayers in 10% FBS /DMEM (GIBCO; #11965-092) at 5% C02. 5075 and GS8-26 primary GBM lines were acquired from the UMASS tissue bank and maintained in defined medium DMEM/F12 1:1, 15 mM/L HEPES, 1X B27 without vitamin A, and supplemented with 20 ng/mL bFGF and EGF [45]. The establishment of the primary line GS8-26 has been presented [45]. The 5075 primary line was prepared in a similar procedure except Liberase instead of trypsin was used to digest the tumor [50]. U87NS and U118NS neurosphere lines were generated from adherent lines, maintained, and passaged as previous described [45].

### Reagents

Temozolomide (T2577) was purchased from Sigma-Aldrich, re-suspended at 10 mg/mL in 100% DMSO, aliquoted, and stored at −20°C. The synthesis and structure of JLK1486 synthesis was previously described [20, 51]. JLK1486 was re-suspended at 10 mM in 100% DMSO, aliquoted, and stored at −20°C.

### MTS assay

The IC50s' of adherent lines was determined by plating 1 × 10^3^ cells/100 uL in 96 well plates and after 24 hours treating the adherent cells with increasing concentrations of JLK1486 (0 μM − 100 μM). Media was aspirated five days later and replaced with CellTiter 96 Aqueous One Solution Cell Proliferation Assay (MTS; Promega G35A) for ~3 hours. Plates were read at A490 nm.

### Primary and secondary neurosphere assays

Neurosphere assays were carried out as previously described [52]. Briefly, U87NS, U118NS, GS8-26, and 5075 lines were pH dissociated, filtered (40 μm), plated at 6,000 cells /2 mL in 6 well plates, and treated with DMSO, JLK1486, TMZ, or TMZ+JLK1486. Primary spheres were counted, fed, and dosed with JLK1486 a second time on day 7 for U87 and U118 and on day 10 for primary lines GS8-26 and 5075. Sphere recovery was determined by counting spheres 7 or 10 days later, day 14 for U87NS and U118NS and day 20 for primary lines. Spheres were then pH dissociated, diluted (U87NS, U118NS: DMSO = 1:100; JLK1486 = 1:50; TMZ = 1:1; TMZ+JLK1486 = 1:1; 5075, GS8-26: DMSO = 1:25; JLK1486 = 1:2; TMZ = 1:2; TMZ+JLK1486 = 1:1), re-plated, and counted 7 or 10 days later, day 21 for U87NS and U118NS and day 30 for 5075 and GS8-26 lines, to determine secondary sphere formation capability.

### Western blotting

Cells were lysed in RIPA Buffer (Boston BioProducts #BP-115), supplemented with protease inhibitor cocktail tablets (Roche, complete mini, #11 836 153 001), and 5 mM NaF. Protein was quantified via Bio-RAD Protein Assay Dye Reagent Concentrate (BIO-RAD, #500-0006) on the Beckman Coulter DU640 Spectrophotometer. Proteins were separated by PAGE and electo-transferred to PVDF membranes (Pall Corporation, BioTrace PVDF 0.45 um, P/N 66543). Membranes were blocked in 5% milk tris-buffered saline with tween 20 (0.1%; TBS-T). Primary antibodies were incubated overnight on a rocker in 5% bovine serum albumin in TBS-T at 1:1000 at 4°C. Membranes were washed the following day 3×, 5′, TBS-T, and incubated with either mouse or rabbit horseradish peroxidase secondary (Cell Signaling #7076S and #7074) for 2 hours room temperature. Proteins were detected via film following the Thermo Scientific's SuperSignal West Pico Chemiluminescent Substrate (Thermo Fisher Scientific, #34087) or Thermo Scientific's SuperSignal West Femto Maximum Sensitivity Substrate (#34095) protocol. The following antibodies were purchased from Cell Signaling Technology: B-Actin (#3700), BiP (#3177), CHOP (#2895), ATF-4 (#11815), Rad51 (#8875), Phospho-Histone H2A.X (#2577), H2A.X (#2595), ATM (#2873), Phospho-ATM (#13050), CHK2 (#6334), Phospho-Chk2 (#2661).

### Trypan blue positive and negative counts

U87NS cells were pH dissociated, filtered, and plated at 250,000/10mL in T75 flasks. Cells were treated with DMSO, JLK1486, TMZ, or TMZ+JLK1486. On day 7 cells were given fresh media and dosed a second time with JLK1486. On day 14 cells were pH dissociated and re-plated at 172,000/10mL in T75 flasks. Cells were pH dissociated and positive and negative trypan blue cells (GIBCO, Trypan Blue Stain 0.4%, #15250) were counted.

### FACS analysis

Drug treated U87NS were pH dissociated, filtered, washed 3X in PBS, and fixed in 95% ethanol overnight at 4°C. Propidium iodide versus Annexin V staining was performed by the UMASS FACS Core, and samples were run on the Calibur FACS machine. Analysis was completed using Flow Jo 7.6

### shRNA ATF4

pGIPZ shATF4 C1 (OligoID: V2LHS_132755), shATF4 E7 (OilgoID: VDLHS_132757), and sh Control were purchased from the UMASS RNAi Core Facility. U87MG cells were infected according to the UMASS RNAi Core Facility Protocol. Briefly, 1 × 10^5^ cells/well were plated, infected 24 hours later with viral supernatant and 1 μg/μl polybrene (Millipore, TR-1003-G), media changed 24 hours post infection and cells were selected for seven days in 2 μg/mL puromycin (GIBCO, #A11138-03). Infected U87MG cells were then converted to U87NS cells as described above.

### Ethics statement

Investigation has been conducted in accordance with the ethical standards and according to the Declaration of Helsinki and according to national and international guidelines and has been approved by the author's institutional review board.

### Mouse xenograft models

Six-week old male NU/NU mice were purchased from Charles River Laboratories and injected with 1 × 10^6^ U87NS cells in 100 uL PBS/right flank. When tumor volumes reached 150 mm^3^ mice were dosed intraperitoneal (IP) with either DMSO (day 0, day3, day 6, day 9), JLK1486 15 mg/kg (day 0, day 7), TMZ 5 mg/kg (day 0, day 7) or with the following dosing regimen: TMZ 5 mg/kg (day 0), JLK 1486 15 mg/kg (day 3), TMZ 5 mg/kg (day 6), JLK1486 15 mg/kg (day 9). Mice were sacrificed when tumors reached 1200 mm^3^.

### H2A.X Immunofluoresence staining

U87NS cells were adhered to slides using Double Cytofunnel Disposable Chambers (Thermo Scientific, #5991039), fixed in 4%PFA/PBS (10 minutes), permeabilized in .5% TRITON X/PBS (5 minutes), blocked in normal goat serum (1 hour), stained overnight with either Phospho-Histone H2A.X (Ser139) (Cell Signaling, #2577) or Rabbit (DA1E) mAb IgG XP Isotype Control (Cell Signaling, #3900), washed in 1X PBS, incubated with goat anti-rabbit IgG (H+L) Secondary Antibody, Alexa Fluor 568 (Life Technologies, #A-11011), and mounted via ProLong Gold Antifade Mountant with DAPI (Molecular Probes by Life Technologies, #P36941). Images were acquired with a Leica wide field scope microscope.

### Statistical analysis *in vitro*

The *t*-test analysis was performed using GraphPad Prism version 6.00 for Mac, GraphPad Software, La Jolla California, USA, www.graphpad.com.

### Statistical analysis *in vivo*

Kaplan-Meier time to tumor volume doubling curves were analyzed via log-rank test.

## SUPPLEMENTARY MATERIALS AND FIGURES


