# Supplementary material for: ER stress in temozolomide-treated glioblastomas interferes with DNA repair and induces apoptosis
Source: Oncotarget. 2016 Jun 7;7(28):43820–34. doi: 10.18632/oncotarget.9907 (PMC5190062; doi:10.18632/oncotarget.9907)
Supplement: Supplementary file 1 [file oncotarget-07-43820-s001.pdf]

ER stress in temozolomide-treated glioblastomas interferes with DNA repair and induces apoptosis

Supplementary Materials

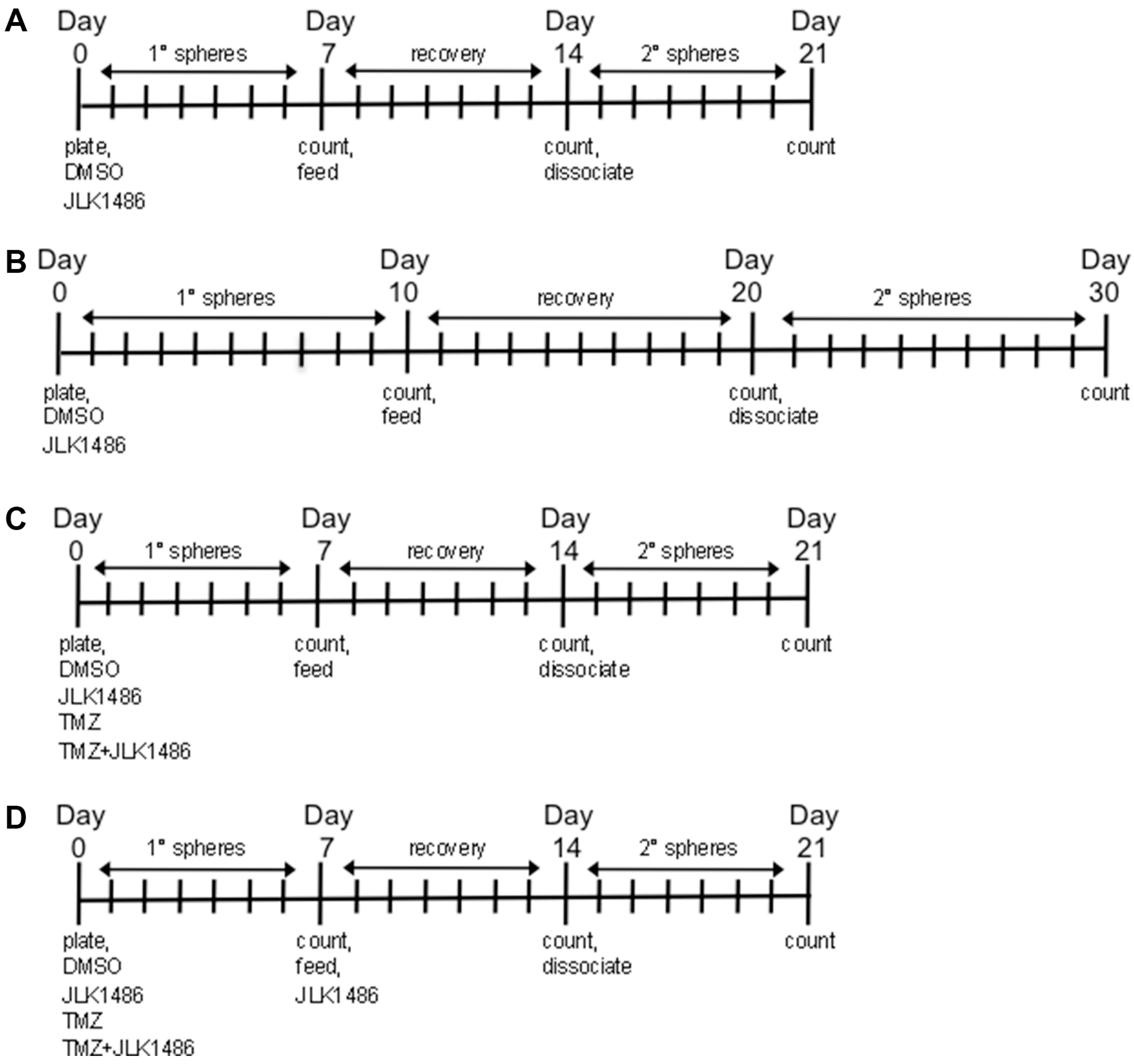

Supplementary Figure S1: Secondary sphere formation timeline for JLK1486 and TMZ+JLK1486 dosing, dissociation, and counting of non-adherent and primary lines. (**A**) Scheme of secondary sphere formation assay depicting timeline of when U87NS and U118NS cell lines were treated with JLK1486 alone, counted, dissociated, and re-counted. (**B**) Scheme of secondary sphere formation assay depicting timeline of when GS8-26 and 5075 primary cell lines were treated with JLK1486 alone, counted, dissociated, and re-counted. (**C**) Scheme of secondary sphere formation assay depicting timeline of when U87NS and U118NS cell lines were treated with both JLK1486 and TMZ. GS8-26 and 5075 primary lines followed scheme shown in panel (B). D. Scheme of secondary sphere formation assay depicting timeline of when U87NS and U118NS cell lines were treated once (1X) with TMZ and twice (2X) with JLK1486. GS8-26 and 5075 primary lines followed scheme shown in panel (B).

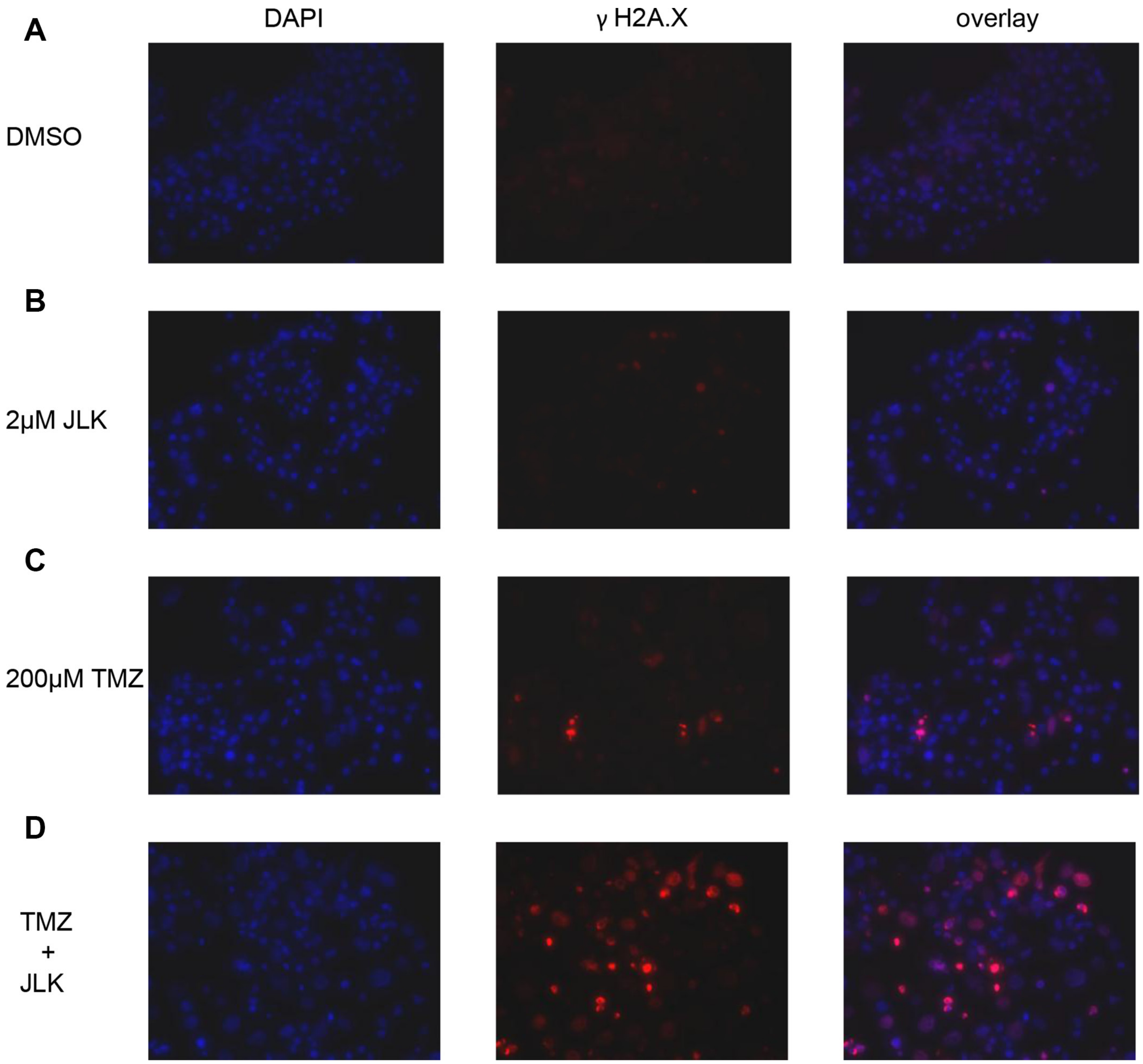

Supplementary Figure S2: Prolonged γH2A.X at day 21 in U87NS TMZ+JLK1486 drug treated cells. (**A**) Day 21 γH2A.X immunofluoresence of DMSO treated U87NS cells. (**B**) Day 21 γH2A.X immunofluoresence of 2 μM JLK1486 treated U87NS cells. (**C**) Day 21 γH2A.X immunofluoresence of 200 μM TMZ treated U87NS cells. (**D**) Day 21 γH2A.X immunofluoresence of TMZ+ 2 μM JLK1486 treated U87NS cells (*N* = 3).

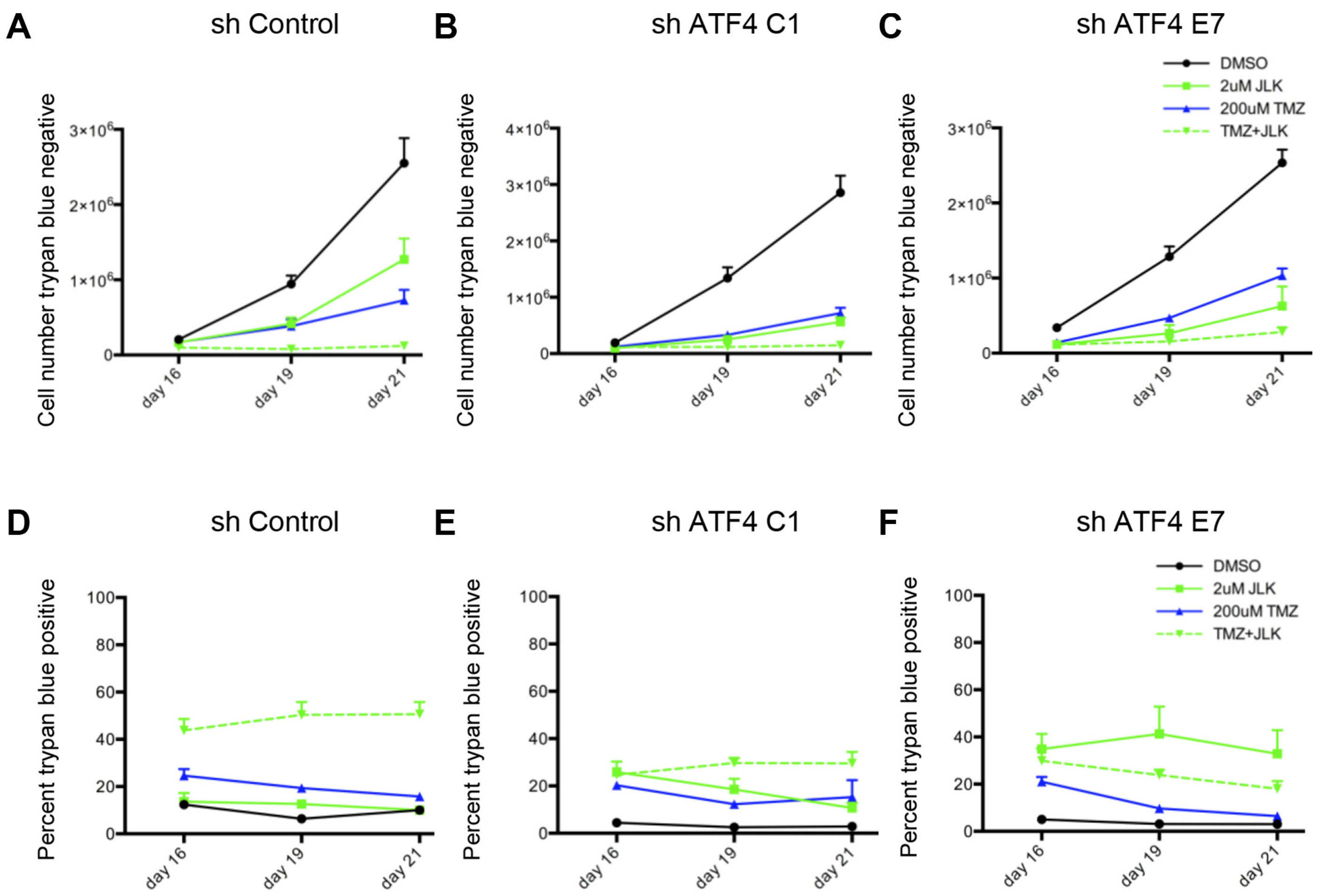

Supplementary Figure S3: Knockdown of ATF4 does not rescue cell growth but does decrease cell death in TMZ+JLK1486 treated U87NS cells. (**A–C**) Trypan blue negative counts at day 16,19, and 21 of U87NS sh Control (A), shATF4 C1 (B), shATF4 E7 (C) cells treated with DMSO, 2μM JLK1486, 200 μM TMZ, or TMZ+ 2 μM JLK1486. (**D–F**) Percent of trypan blue positive cells at day 16, 19, and 21 of U87NS sh Control (D), shATF4 C1 (E), shATF4 E7 (F) cells treated with DMSO, 2 μM JLK1486, 200 μM TMZ, or TMZ+ 2 μM JLK1486 (*N* = 3), SEM.
